# Non-traditional stable isotope behaviors in immiscible silica-melts in a mafic magma chamber

**DOI:** 10.1038/srep17561

**Published:** 2015-12-01

**Authors:** Dan Zhu, Huiming Bao, Yun Liu

**Affiliations:** 1 State Key Laboratory of Ore Deposit Geochemistry, Institute of Geochemistry, Chinese Academy of Sciences, Guiyang 550081, China; 2 Department of Geology and Geophysics, E235 Howe-Russell Complex, Louisiana State University, Baton Rouge, LA 70803, USA

## Abstract

Non-traditional stable isotopes have increasingly been applied to studies of igneous processes including planetary differentiation. Equilibrium isotope fractionation of these elements in silicates is expected to be negligible at magmatic temperatures (δ^57^Fe difference often less than 0.2 per mil). However, an increasing number of data has revealed a puzzling observation, e.g., the δ^57^Fe for silicic magmas ranges from 0‰ up to 0.6‰, with the most positive δ^57^Fe almost exclusively found in A-type granitoids. Several interpretations have been proposed by different research groups, but these have so far failed to explain some aspects of the observations. Here we propose a dynamic, diffusion-induced isotope fractionation model that assumes Si-melts are growing and ascending immiscibly in a Fe-rich bulk magma chamber. Our model offers predictions on the behavior of non-traditional stable isotope such as Fe, Mg, Si, and Li that are consistent with observations from many A-type granitoids, especially those associated with layered intrusions. Diffusion-induced isotope fractionation may be more commonly preserved in magmatic rocks than was originally predicted.

Non-traditional stable isotopic compositions in terrestrial and extraterrestrial igneous rocks have become an increasingly useful tool in studying processes that govern planet formation^1^, core formation^2^, mantle melting^3^, magmatic differentiation^4^, mantle heterogeneity^5^ and genesis of ore deposit^6^. Variations in stable isotope ratios are often in the parts per thousand range and therefore are generally reported as permil variations from a reference material most commonly in δ-notation^7^. The mechanism of isotope fractionation can be interpreted based on equilibrium and kinetic thermodynamics^8^,^9^. The former is expected to generate small degrees of fractionation at magmatic temperatures. For example, magmatic differentiation processes associated with MORBs and OIBs result in δ^57^Fe ranging within 0.12‰, and 0.15‰, respectively^10^, and δ^26^Mg values of both MORBs and OIBs ranging within 0.1‰^3^. Often, Mg isotopes barely fractionate during magmatic differentiation, e.g. the formation of I-type granitoids in the Lachlan Fold Belt^11^. In Hawaiian basalts Mg isotope composition does not display significant changes either^3^,^12^, although the range of δ^57^Fe can be larger than 0.2‰, which has been attributed to the increase of Fe ^3+^/Fe _total_ ratios in basalts during olivine crystallization^4^.

It has been noted, however, that the δ^57^Fe value for silicic rocks ranges from 0‰ up to 0.6‰^13^,^14^,^15^, with the most positive δ^57^Fe being almost exclusively found in A-type granitoids^15^,^16^,^17^. In fact, there is a general trend among A-type felsic rocks of increasing δ^57^Fe values associated with increasing SiO_2_ content^15^,^16^,^17^. A-type felsic rocks often occur at the top of many layered intrusions and many tholeiitic provinces, and are interpreted to be the products of magmatic differentiation^18^,^19^,^20^,^21^.

The unusual enrichment of heavy Fe isotopes in A-type granitoids is puzzling and has been the subject of intense debate in the community. It has been attributed to the separation of magma under a temperature gradient or the Soret effect^22^,^23^. However, the Soret effect cannot explain the absence of isotope fractionation in U^22^ or Li (this study) in A-type granitoids as these isotopes have displayed significant Soret diffusional isotope fractionation in the laboratory^24^,^25^. Another attempt to explain the enrichment of heavy Fe isotopes in some A-type granitoids invokes fluid exsolution, i.e. a fluid in equilibrium with a magnetite-bearing magma is removed from a system^13^,^14^. However, the lack of isotope fractionation for the equally fluid-mobile Zn does not support such mechanism^16^. In addition, fluid loss would result in decreasing δ^30^Si with increasing SiO_2_ content, a trend opposite to what has been observed^23^. Magmatic differentiation or fractional crystallization^4^,^26^ as alternative mechanisms have been extensively reviewed^16^,^17^,^23^, and it was concluded that this mechanism is unsatisfactory because modeled trends are opposite to the observed δ^57^Fe trend in A-type granite melts^16^,^23^. Another recently proposed interpretation invokes Fe’s redox behavior in magmatic processes^15^,^17^. However, this mechanism does not explain the concurrent enrichment of Mg isotopes in A-type granitoids because the redox mechanism would predict no effect on Mg isotope composition (Telus, *et al.*^16^ and references therein). The mean force constant calibration for bulk Fe in magmas is another recent attempt in improving understanding of the equilibrium Fe isotope effect in igneous rocks^27^. However, the extent of equilibrium Fe isotope fractionation brought on by a change in the force constant at high temperatures is limited. As pointed out by the authors, a sudden increase in the force constant of bulk Fe from dacite (SiO_2_ > 61 wt.%) to rhyolite (SiO_2_ > 69 wt.%) could explain about 1/3 of the δ^56^Fe difference between MORBs and their source; it cannot explain the whole range of Fe isotope fractionation in A-type granitoids. Importantly, the same bulk Fe force constant difference would predict a similar enrichment of heavy Fe isotopes in I- and S-type granitoids as well, a prediction that is not consistent with observations. I- and S-type granitoids are in fact systematically lighter in δ^57^Fe (<0.4‰)^15^,^16^. Finally, the reservoir effect related to equilibrium fractionation processes (i.e. a Rayleigh process) may potentially generate unusually large ^57^Fe enrichments, such as those in A-type granitoids. However, a Rayleigh model shows that A-type granite melts should become isotopically lighter as magnetite, a mineral phase favoring heavy Fe isotopes, is removed from the melts progressively, a trend that is opposite to the observed δ^57^Fe trend^23^.

Chemical diffusion can lead to a diffusional or diffusion-induced isotope effect (DIE), which is a well-known phenomenon in liquids^28^. Since a heavier isotope has a slightly lower diffusion coefficient than a lighter isotope of the same element, the heavier isotope will lag behind as both diffuse through a chemical potential gradient. Thus, during the growth of a crystal or an immiscible liquid, the heavier Fe isotope is expected to be relatively enriched at the high end of a concentration gradient, i.e., at the interfacial melt (Fig. 1). The DIE in magma can be large as shown by experiments^29^,^30^,^31^. Therefore it is reasonable to assume that δ^57^Fe larger than 0.2‰ isotopic fractionations in natural igneous systems may originate from DIE. Although DIE has been documented in natural olivines^32^,^33^ and intrusion boundaries^34^, the natural occurrence of DIE in the molten state of a magmatic system has not been recognized. Here we propose that under a condition when Si-melts ascend, immiscibly, from a bulk Fe-rich magma, signals of element-specific DIE are preserved in felsic rocks such as the A-type granitoids. In this study, we first examine the growth dynamics of ascending Si-melts, and then quantitatively predict the isotope behaviors of Fe, Mg, Si, and Li under these given conditions so as to shed light on some of the non-traditional stable isotope observations in igneous systems.

## Model

**Figure 1:**
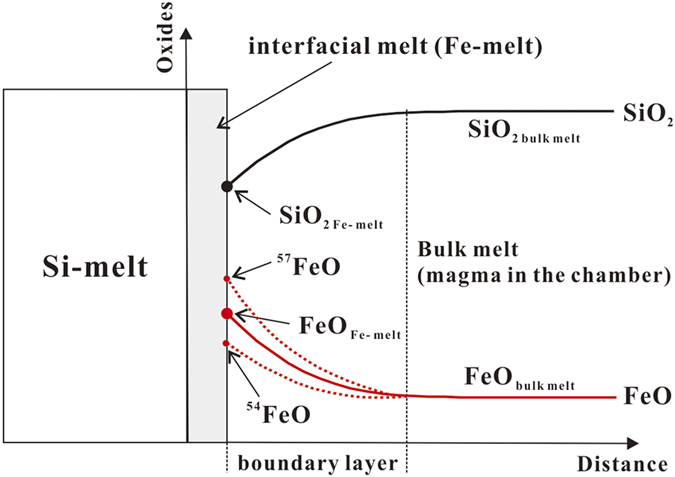
Schematic illustration of FeO and SiO_2_ compositional profile and diffusion-induced Fe isotope fractionation of interfacial melt during the convective growth of an ascending Si-melt. ^57^FeO or ^54^FeO line represents a hypothetical diffusional profile for the corresponding pure FeO isotope endmember. The thickness of the interfacial melt is exaggerated for illustration purpose. See text for details.

Silica liquid immiscibility was suggested as a method of magmatic differentiation a century ago^35^. However, it is only recently, thanks to progress in experimental and petrographic studies^36^, that A-type felsic rocks at the top of many layered intrusions and in many tholeiitic provinces have been regarded as immiscible silica melts^18^,^19^,^20^,^21^. The process of silica liquid immiscibility is complex and not well understood and is beyond the scope of the present study. Here we only address the isotopic behavior of immiscible Si-melts in a mafic magma. Once nucleated, Si-melts, will grow at the expense of the bulk melt. The less dense Si-melts rise toward the top of the magma chamber. The growth rate will depend on the position of the bulk magma within the Si-liquid and Fe-liquid field. A schematic illustration of the FeO and SiO_2_ compositional profile during a convective growth of ascending Si-rich melts is presented in Fig. 1.

According to the spinodal diagram of tholeiitic basalt, the interfacial melt of a growing Si-melt equilibrates chemically with a Fe-melt. The growth rate of immiscible Si-melt is determined by the compositional difference between its interfacial melt (Fe-melt) and the bulk melt^37^ (Fig. 1). For example, if we assume that SiO_2_ is the principal equilibrium-determining component for the Si-melt^38^, the growth rate of a Si-melt is determined by ∆(SiO_2 bulk melt_ –SiO_2 Fe-melt_), where SiO_2 bulk_ and SiO_2 Fe-melt_ are silica contents of the bulk melt and the interfacial Fe-melt, respectively.

Under this growth scenario, a local thermodynamic equilibrium between the growing Si-melt and its interfacial melt (Fe-melt) holds^39^. Therefore, the isotopic composition of a growing Si-melt is equal to its interfacial melt (the Fe-melt) if we ignore the small equilibrium fractionation effect^40^. According to Fick’s first law, the diffusive flux of an element for a growing Si-melt is


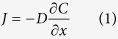


which can be rewritten as


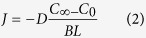


where *J* is the diffusive flux, *D* is the diffusion coefficient, *C* is the concentration, *x* is distance, *∂C/∂x* is the concentration gradient, *C*_*∞*_ is the concentration in the far-field (magma in the chamber), *C*_*0*_ is the interfacial melt concentration, and *BL* is thickness of the compositional boundary layer. Due to the difference in diffusion coefficient among isotopes of an element, the compositional difference in isotopes at the interface (∆*C*) can be expressed as


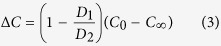


where *D*_*1*_ and *D*_*2*_ are diffusion coefficients for heavy and light isotope, respectively. For example, the diffusivity (*D*_*1*_) of the heavy isotope ^57^Fe is less than that (*D*_*2*_) of the lighter ^54^Fe, therefore the Si-melt becomes enriched in ^57^Fe (Fig. 1). Ignoring the small equilibrium fractionation factor for isotopes at high temperatures, we can obtain a solution for a steady-state isotope composition of a crystal growing in an infinite medium:


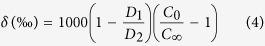


where *δ* is the difference between isotope ratio in a crystal or a liquid and of the bulk magma (in ‰) following Watson and Miller^40^. Since *D*_*1*_/*D*_*2*_ = (*m*_*2*_/*m*_*1*_)β^41^, equation 4 can be rewritten as


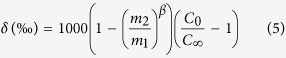


where *m*_*1*_ and *m*_*2*_ are masses of heavy and light isotopes, respectively. *β* is an empirical parameter obtained from experiments^29^ and theoretical computation^42^.

The isotopic compositions of a major element of a growing Si-melt can be expressed as


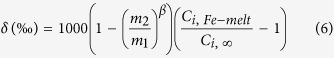


where *C*_*i*_,_*∞*_ is the concentration of element *i* in the bulk melt, *C*_*i, Fe-melt*_ is the concentration of element *i* in the interfacial melt, i.e., Fe-melt.

Diffusion of trace elements is often complicated by processes such as: (1) the fact that interface-melt concentration is not fixed by thermodynamic equilibrium, and (2) uphill diffusion^37^ (diffusion of a component against its concentration gradient caused by decoupling of concentration and chemical activity of an element^43^). Therefore, the isotope behavior of a trace element cannot be represented simply by Eq. (6). Here we ignore multicomponent effects and treat trace element diffusion as binary. We obtain the interfacial concentration for a growing Si-melt according to Zhang^37^


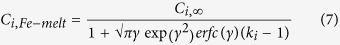


where *C*_*i,∞*_ is the concentration of trace element *i* in the far-field melt (magma in the chamber); *C*_*i,Fe-melt*_ is the interfacial melt concentration of trace element *i*, *erfc* is the complimentary error function, *k*_*i*_ is simple partition coefficient of element *i* between crystal and melt; *γ* = *α*(*D/D*_*i*_)^1/2^, where *D* and *D*_*i*_ are diffusion coefficients of the major component and trace element *i*, respectively; α is a parameter related to composition or growth rate and is determined by the major component, for example, SiO_2_ in a Si-melt, and can be solved by


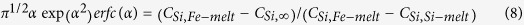


where *C*_*Si, Fe-melt*_, *C*_*Si, ∞*_and *C*_*Si, Si-melt*_ are the concentration of SiO_2_ in the Fe-melt, bulk melt and Si-melt, respectively. Solving Eq. (6) for *δ* for a minor or trace element yields


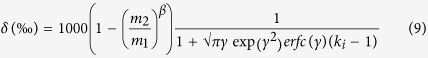


The above solution shows that the isotope fractionation for a trace element is independent of its own concentration and is controlled by the growth rate of a crystal or immiscible liquid (equivalent to *α*), its partition coefficient, and its diffusivity.

### Model predictions

Now we have quantitative models for isotope behavior of both major and trace elements in immiscibly growing Si-melts. We now examine two major elements Fe and Si and two trace elements Mg and Li.

**Figure 2:**
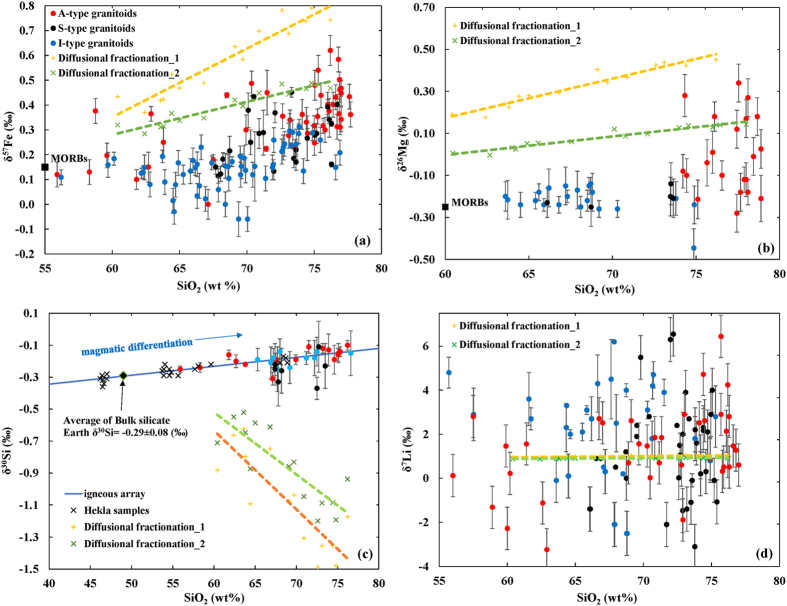
Observed and predicted stable isotope compositions of granitoids. (**a**) Fe isotopes from Telus, *et al.*^16^, Sossi, *et al.*^17^, Zambardi, *et al.*^23^, Foden, *et al.*^15^ and references therein (compiled in Supplementary Information). The δ^57^Fe value of terrestrial basalts (MORBs) is from Teng, *et al.*^10^. The diffusional fractionation trend 1 and 2 (orange and green dash lines) are calculated using Eq. (6) based on data reported in Table 1. (**b**) Mg isotopes from Telus, *et al.*^16^ and references therein (Supplementary Information). The δ^26^Mg value of terrestrial basalts (MORBs) is from Teng, *et al.*^3^. The diffusional fractionation trend 1 and 2 (orange and green dash lines) are calculated using Eq. (9) based on data reported in Table 2. (c) Plot of δ^30^Si vs. SiO_2_ displaying an “igneous array” (blue line) for Si isotopes from Savage, *et al.*^46^, Savage, *et al.*^59^, and Zambardi, *et al.*^23^ (Supplementary Information). The diffusional fractionation trend 1 and 2 (orange and green dash lines) are calculated using Eq. (9) based on data reported in Table 3. (**d**) Li isotope data from Li, *et al.*^11^ and Teng, *et al.*^60^ (Supplementary Information). The diffusional fractionation trend 1 and 2 (orange and green dash lines) are calculated using Eq. (9) based on data reported in Table 4.

Applying Eq. (6) we have calculated the Fe isotope effect using published experimental data by Charlier and Grove^39^, a *β* value of 0.015^41^, an initial δ^57^Fe value of δ^57^Fe_MORBs_, and a *C*_*Fe*_,_*∞*_ value taken as (*C*_*Fe-liquid*_ + *C*_*Si-liquid*_)/2 and (2/3× *C*_*Fe-liquid*_ + 1/3 ×*C*_*Si-liquid*_), respectively (Table 1). Because the concentration of interface Fe is always higher than that of the far-field magma during the growth of a Si-melt, i.e., *C*_*Fe, Fe-melt*_ > *C*_*Fe*_,_*∞*_, the first two terms on the right hand side of Eq. (6) are both positive. Thus, qualitatively, the Si-melt will always be enriched in heavy Fe isotopes (Fig. 1). The calculated result shows that the δ (Si-liquid-bulk magma) increases with increasing SiO_2_ content in the Si-melt (Fig. 2a). Comparing with the Fe isotope data of igneous systems published in recent years (Fig. 2a), the unusually heavy Fe isotope enrichment in some of the Si-rich A-type granitoids is consistent with the model predictions.

Examining the data further, it is apparent that the calculated δ^57^Fe, assuming a far-field Fe concentration *C*_*Fe*_,_*∞*_ = 1/2× *C*_*Fe-liquid*_ +1/2 ×*C*_*Si-liquid*_ (orange dash line in Fig. 2a), is higher than those observed in A-type granitoids. However, when the *C*_*Fe*_,_*∞*_ value is taken as “2/3× *C*_*Fe-liquid*_ + 1/3 ×*C*_*Si-liquid*_” (green dash line in Fig. 2a), the observed data match with our modeled ones very well. Additional factors may play a role for values lower than the model-predicted δ^57^Fe values for SiO_2_% higher than 70% (Fig. 2a). For example, there may be some degree of isotope re-equilibrium between the Si-liquid and the bulk melt. In other words, if a Si-liquid cannot be separated from the rest of magma effectively, it will be isotopically homogenized, such as is likely for the Skaergaard intrusion^44^. Also, some A-type granitoids were not formed by the process envisioned by our model but rather by fractional crystallization and re-melting of tholeiitic material^45^, these two process are unlikely to produce the observed Fe isotope fractionation in A-type granitoids, as suggested^16^,^23^.

Si is also a major element in melts. According to Eq. (6), qualitatively, the δ should be opposite to that of Fe and the Si-melts should be enriched in light Si isotopes because *C*_*Si, Fe-melt*_ < *C*_*Si*_,_*∞*_ in the right-hand term of the equation. In fact, the ratio of *C*_*Si, Fe-melt*_ /*C*_*Si*_,_*∞*_ in Si-melts produced by experiments can range from 0.67 to 0.92, if we use the same published experimental data^39^ and bulk *C*_*Si*_,_*∞*_ = (C_*Si*_, _*Fe-melt*_ + C_*Si*_, _*Si-melt*_)/2, the calculated δ^30^Si ranges from −0.52 to −1.49‰ (Table 2) if we use a Si *β* factor of 0.047^42^. However, experiments have yielded a near-zero *β* value for Si isotopes during chemical diffusion^29^, which is very different from 0.047, a value obtained from classical molecular dynamics calculations of a simple SiO_2_-MgO system^42^. One possible explanation for the observed near-zero *β* value is that the diffusing species of Si is a network former and diffuses as a large species, e.g., as [SiO_4_] _n_. If *β* is near-zero, the δ will be close to zero regardless the value of *C*_*Si, Fe-melt*_/*C*_*Si*_,_*∞*_ in the second term of Eq. (6). Therefore, diffusional enrichment of lighter Si isotopes in Si-melts should be negligible.

Indeed, the observed pattern is different between Si isotopes and Fe isotopes. The δ^30^Si –[SiO_2_]% plot displays a positive correlation that is shared by A-, I-types of granitoids and basalts (Fig. 2c), and by samples of different locations with distinctly different mineral assemblages, such as Hekla^46^ and Cedar Butte volcano^23^. This can be explained by the equilibrium silicate melt structure being an overwhelming control on Si isotope composition, see Fig. 4 in Zambardi, *et al.*^23^. Although the equilibrium Si fractionation factor between two conjugate immiscible silicate melts has not been calculated or measured, qualitative evidence indicates that heavier Si isotopes are enriched in the more polymerized melts, i.e. heavier Si isotopes increase as the ratio of NBO (non-bridging oxygen) to T (tetrahedron) decreases (Fig. 2c). It is possible that, at equilibrium, bonding with a BO (bridging oxygen) prefers slightly heavier Si isotopes than bonding with NBO in silicate melts. This feature is consistent with the fact that ^18^O is also preferred in the immiscible Si-melts, the more polymerized structure melt, a phenomenon observed in experiments^47^,^48^.

The S-type granitoids are slightly enriched in light Si isotopes with respect to I- and A-type granitoids (Fig. 2c) because the main source of S-type granitoids is sediments^49^ which are commonly enriched in light Si isotopes relative to igneous rocks^50^. Overall, diffusion does not seem to play any significant role in Si isotope distribution during magmatic processes.

In A-type granitoids, Mg can be treated as a trace element whose chemical properties are similar to Fe during silicate melt unmixing^39^. Thus Mg’s isotope behavior should be similar to Fe’s. Indeed, our calculation using Eq. (9) under the same magmatic conditions shows that δ^26^Mg increases with increasing SiO_2_ content in ascending Si-melts (Table 3), a prediction in close agreement with the observed trend (Fig. 2b). The reasons for the calculated values being higher than the observed ones are similar to the reasons given for Fe isotopes.

Li is a trace element known to have a high diffusivity in melts. According to Eq. (9), at a high diffusion rate, γ (γ = *α*(*D*_*Si*_*/D*_*Li*_)^1/2^) approaches zero because *D*_*Li*_ ≫ *D*_*Si*_, which leads the second term on the right to approach zero as well, resulting in a near-zero δ value (Table 4). So far, observed data do not display any correlation between δ^7^Li and [SiO_2_]% or among A-type, I-type, and S-type granitoids (Fig. 2d). This is consistent with our model prediction for any element with a high diffusivity. The observed large spread of Li isotope composition of A-type granitoids must be due to other processes.

It is worth noting that our model treats the bulk magma as an infinite reservoir for Fe and Mg. The rationale for this is: (1) Experiments have shown that the evolved silicate melt produced by fractional crystallization from a MORB basalt just prior to silicate melt unmixing is less than 30% of the total volume of the MORB basalt^51^ and the Fe-rich melt is constantly exchanging with the 70% precipitated minerals. (2) Evolved melt prior to unmixing is extremely enriched in FeO and is similar in composition to immiscible Fe-melt^51^. The volume of the immiscible Si-melt is thus less than that of its conjugate Fe-melt according to the lever rule. (3) The amount of Fe and Mg “extracted” by the immiscible Si-melt is limited, because the Fe and Mg concentrations in Si-melt are 2-4 times less than those in Fe-melt^39^,^51^. Therefore, DIE has little impact on the Fe and Mg isotopic compositions of the far-field magma. Since our model shows that Si and Li do not have measurable DIE during the immiscible ascension of silicate melts in mafic magma the reservoir effect or mass-balance is not an issue.

### Further predictions

As predicted above, the isotope behavior of Fe, Mg, Si, and Li in an immiscible and growing Si-melts can be more or less determined by elemental concentration, growth rate of the immiscible liquid, the partition coefficient, and diffusivity. Unfortunately, non-traditional stable isotope data for A-type granitoids are still sporadic in the literature at this time. For example, Fe or Mg isotope data for A-type granitoids with <70 wt.% SiO_2_ are rare (Figs 2a,b). Filling this data gap with future work should present a test of our model for natural silicate melts at higher temperatures. In addition, non-traditional stable isotope data for A-type granitoids at the top of large layered intrusions, e.g. the Bushveld Complex can be used to test if immiscibility did occur in those intrusions^18^. Furthermore, Fe or Mg isotope compositions can be used to distinguish the origin of different varieties of A-type granites. For example, A-type granites can also form via extreme fractional crystallization of a basaltic magma or partial melt of a basaltic parent rock^45^. In this case the Fe and Mg isotope compositions will be controlled by equilibrium isotope effects which generate smaller degrees of isotope fractionation than diffusion induced isotope effects associated with Si-melts formed through immiscibility.

Apart for the aforementioned isotope systems, our model is consistent with Zn and Mo isotope behaviors in Hekla rhyolitic melts^52^,^53^. Although the *β* parameters for Zn and Mo have not been determined by experiments, the isotope behaviors of Zn in Hekla rhyolitic melts should be similar to those of Fe and Mg considering the association of Zn with Fe-melts^54^ and its similar atomic weight with Fe. According to Richter, *et al.*^29^, the atomic weight of element Mo is too large to have a sizeable *β* value. Therefore Mo isotope fractionation in Hekla rhyolitic melts should be absent, as has been observed^53^. Our calculated DIE for Zn and Mo with these assumptions can be found in Fig. S1 in Supplementary Information. Our model also gives a testable prediction on isotope behaviors for other systems in A-type granitoids. For example, we predict a large DIE for Ti during silicate melt unmixing due to the larger ^50^Ti/^46^Ti mass ratio and an expected larger *β* value for Ti^29^. We also predict that highly compatible elements in immiscible Si-melts, e.g. K^54^, should have an isotopic pattern opposite to those of Fe and Mg. Nevertheless, similar to Li, the high diffusion rate of K in a basalt^55^ may result in little to no apparent isotope fractionation. In addition, experimental results indicated that diffusion-induced Ca isotope fractionation depends on silicate liquid’s composition^30^. This composition-dependent DIE is not fully understood at a molecular level. Our model, combined with the large variations in chemical compositions of the A-type granitoids, may shed light on the puzzling Ca isotope behavior in melts during silicate melt unmixing.

While our predictions await testing by new isotope measurements, we would like to point out one broader implications of the immiscibility-based model. Other processes, such as bubble growth in melts or liquids^56^ and carbonatite genesis^57^ are controlled by immiscibility. Diffusional isotope effects in erupted volcanic gases or in carbonatites could bear information on the dynamics of igneous processes, as has already been speculated^58^.

## Additional Information

**How to cite this article**: Zhu, D. *et al.* Non-traditional stable isotope behaviors in immiscible silica-melts in a mafic magma chamber. *Sci. Rep.* **5**, 17561; doi: 10.1038/srep17561 (2015).

## Supplementary Material

Supplementary Information

## Figures and Tables

**Table 1:** Calculated results of diffusional fractionation of Fe isotopes in immiscible Si-melts.

Run	T (°C)	SiO_2_	C_0_	C_c_	*β*_FeO_	C∞ _(1/2+1/2)_	δ^57^Fe_(1/2+1/2)_	C∞ _(2/3+1/3)_	δ^57^Fe_(2/3+1/3)_
SI-13	1020	60.4	21.40	10.30	0.015	15.85	0.43	17.70	0.32
SI-7	1006	69.1	23.10	5.79	0.015	14.45	0.64	17.33	0.42
SI-5	1006	64.4	24.60	9.06	0.015	16.83	0.52	19.42	0.37
SI-8	963	70.9	28.10	5.43	0.015	16.77	0.70	20.54	0.45
SI-9	938	72.6	30.60	3.79	0.015	17.20	0.78	21.66	0.48
M-9	1020	63.8	21.80	10.80	0.015	16.30	0.42	18.13	0.31
M-4	1006	73.1	25.80	5.21	0.015	15.51	0.69	18.94	0.44
M-5	1005	69.7	25.20	7.62	0.015	16.41	0.58	19.34	0.40
M-6	963	74.4	32.00	5.09	0.015	18.55	0.74	23.03	0.47
M-7	938	74.8	32.40	3.79	0.015	18.10	0.79	22.86	0.49
I-3	1005	62.4	21.80	12.50	0.015	17.15	0.37	18.70	0.28
I-5	964	76.2	27.60	4.27	0.015	15.94	0.74	19.82	0.47
S-6	1023	65	19.40	8.45	0.015	13.93	0.47	15.75	0.34
S-3	1005	63.6	18.20	9.14	0.015	13.67	0.42	15.18	0.31
S-5	1006	66.8	18.90	7.77	0.015	13.34	0.49	15.19	0.35

Note: the first three columns in Table 1 to Table 6 are the same, which are experimental data from Charlier and Grove^39^. SiO_2_ is the SiO_2_ concentration of immiscible Si-melt. C_0_: FeO concentration of interfacial melt (Fe-melt). C_c_: FeO concentration of Si-melt. *β*_FeO_: FeO diffusional fractionation factor is from Richter *et al.* (2009). C_∞ (1/2+1/2)_: FeO concentration of the bulk melt = 1/2× C_0_ + 1/2× C_c_. δ^57^Fe _(1/2+1/2)_: calculated δ^57^Fe with the bulk melt = C∞ _(1/2+1/2)_, and initial δ^57^Fe = δ^57^Fe_MORBs_ is assumed. C_∞ (2/3+1/3)_: FeO concentration of the bulk melt = 2/3× C_0_ +1/3× C_c_. δ^57^Fe _(2/3+1/3)_: calculated δ^57^Fe with the bulk melt = C∞ _(2/3+1/3))_, and initial δ^57^Fe = δ^57^Fe_MORBs_ is assumed.

**Table 2:** Calculated results of diffusional fractionation of Si isotopes in immiscible Si-melts.

Run	T (°C)	SiO_2_ (C_c_)	C_0_	*β*_SiO2_	C_∞ (1/2+1/2)_	δ^30^Si_(1/2+1/2)_	C_∞ (2/3+1/3)_	δ^30^Si_(2/3+1/3)_
SI-13	1020	60.4	41.7	0.047	51.05	−0.88	47.93	−0.71
SI-7	1006	69.1	42.1	0.047	55.60	−1.08	51.10	−0.86
SI-5	1006	64.4	38.8	0.047	51.60	−1.09	47.33	−0.87
SI-8	963	70.9	37	0.047	53.95	−1.31	48.30	−1.05
SI-9	938	72.6	33.4	0.047	53.00	−1.49	46.47	−1.20
M-9	1020	63.8	46.5	0.047	55.15	−0.80	52.27	−0.65
M-4	1006	73.1	36.9	0.047	55.00	−1.36	48.97	−1.09
M-5	1005	69.7	43.5	0.047	56.60	−1.04	52.23	−0.83
M-6	963	74.4	37.6	0.047	56.00	−1.35	49.87	−1.09
M-7	938	74.8	34.6	0.047	54.70	−1.48	48.00	−1.19
I-3	1005	62.4	49.5	0.047	55.95	−0.66	53.80	−0.55
I-5	964	76.2	43.5	0.047	59.85	−1.17	54.40	−0.94
S-6	1023	65	49.9	0.047	57.45	−0.72	54.93	−0.59
S-3	1005	63.6	51.7	0.047	57.65	−0.62	55.67	−0.52
S-5	1006	66.8	50.2	0.047	58.50	−0.75	55.73	−0.61

Note: C_0_: SiO_2_ concentration of interfacial melt (Fe-melt). *β*_SiO2_: SiO_2_ diffusional fractionation factor is from Goel, *et al.*^42^. C_∞ (1/2+1/2)_: SiO_2_ concentration of the bulk melt = 1/2× C_0_ + 1/2× C_c_. δ^30^Si _(1/2+1/2)_: calculated δ^30^Si with the bulk melt = C_∞ (1/2+1/2)_, and initial δ^30^Si = −0.29 is assumed according to Savage, *et al.*^46^. C_∞ (2/3+1/3)_: SiO_2_ concentration of the bulk melt=2/3× C_0_ + 1/3× C_c_. δ^30^Si _(2/3+1/3)_: calculated δ^30^Si with the bulk melt = C∞_(2/3+1/3)_.

**Table 3:** Calculated results of diffusional fractionation of Mg isotopes in immiscible Si-melts.

Run	T (°C)	SiO_2_	*β*_MgO_	NBO/T_si-melt_	K_Mg_	D_Mg_/D_si_	*γ*1	α_(1/2+1/2)_	δ^26^Mg_(1/2+1/2)_	*γ*2	α_(2/3+1/3)_	δ^26^Mg_(2/3+1/3)_
SI-13	1020	60.4	0.045	0.45	1.71	5.76	0.18	0.433	0.19	0.10	0.242	0.01
SI-7	1006	69.1	0.045	0.19	2.46	6.02	0.18	0.433	0.40	0.10	0.242	0.12
SI-5	1006	64.4	0.045	0.32	1.97	6.02	0.18	0.433	0.28	0.10	0.242	0.05
SI-8	963	70.9	0.045	0.18	2.50	6.97	0.16	0.433	0.37	0.09	0.242	0.10
SI-9	938	72.6	0.045	0.12	3.06	7.62	0.16	0.433	0.43	0.09	0.242	0.13
M-9	1020	63.8	0.045	0.40	1.79	5.76	0.18	0.433	0.23	0.10	0.242	0.02
M-4	1006	73.1	0.045	0.16	2.63	6.02	0.18	0.433	0.44	0.10	0.242	0.14
M-5	1005	69.7	0.045	0.25	2.21	6.04	0.18	0.433	0.34	0.10	0.242	0.09
M-6	963	74.4	0.045	0.14	2.84	6.97	0.16	0.433	0.42	0.09	0.242	0.13
M-7	938	74.8	0.045	0.10	3.27	7.62	0.16	0.433	0.45	0.09	0.242	0.14
I-3	1005	62.4	0.045	0.46	1.69	6.04	0.18	0.433	0.18	0.10	0.242	0.00
I-5	964	76.2	0.045	0.12	3.02	6.95	0.16	0.433	0.45	0.09	0.242	0.14
S-6	1023	65	0.045	0.33	1.94	5.70	0.18	0.433	0.28	0.10	0.242	0.05
S-3	1005	63.6	0.045	0.37	1.87	6.04	0.18	0.433	0.24	0.10	0.242	0.03
S-5	1006	66.8	0.045	0.30	2.03	6.02	0.18	0.433	0.29	0.10	0.242	0.06

Note: *β*_Mg_: Mg diffusional fractionation factor is from Richter, *et al.*^41^. NBO/T_si-melt_: the ratio of NBO (non-bridging oxygen) to T (tetrahedron) of the Si-melt. K_Mg_: partition coefficient of Mg between two immiscible silicates melt. K_Mg_ = 1.2129(NBO/T_si-melt_)^−0.428^ is obtained by fitting experimental data of Veksler, *et al.*^54^. D_Mg_/D_si_: ratio of diffusivity of Mg over Si in basaltic melt is from Zhang, *et al.*^55^. α _(1/2+1/2)_: calculated α value with the bulk melt = C_∞ (1/2+1/2)_. *γ*1: calculated *γ* value using α _(1/2+1/2)_. δ^26^Mg _(1/2+1/2)_: calculated δ^26^Mg with the bulk melt = C_∞ (1/2+1/2)_, and initial δ^26^Mg = δ^26^Mg_MORBs_ is assumed according to Teng, *et al.*^3^. α _(3/3+1/3)_: calculated α value with the bulk melt = C_∞ (2/3+1/3)_. γ2: calculated *γ* value using α _(3/3+1/3)_. δ^26^Mg _(2/3+1/3)_: calculated δ^26^Mg with the bulk melt = C_∞ (2/3+1/3)_.

**Table 4:** Calculated results of diffusional fractionation of Li isotopes in immiscible Si-melts.

Run	T (°C)	SiO_2_	*β*_Li_	NBO/T_si-melt_	K_Li_	D_Li_/D_si_	*γ*1	α_(1/2+1/2)_	δ^7^Li_(1/2+1/2)_	*γ*2	α_(2/3+1/3)_	δ^7^Li_(2/3+1/3)_
SI-13	1020	60.4	0.215	0.45	1.35	1605.15	0.01	0.433	1.16	0.01	0.242	1.09
SI-7	1006	69.1	0.215	0.19	1.84	1863.27	0.01	0.433	1.26	0.01	0.242	1.15
SI-5	1006	64.4	0.215	0.32	1.53	1863.27	0.01	0.433	1.20	0.01	0.242	1.11
SI-8	963	70.9	0.215	0.18	1.87	3008.58	0.01	0.433	1.21	0.00	0.242	1.12
SI-9	938	72.6	0.215	0.12	2.22	4037.70	0.01	0.433	1.22	0.00	0.242	1.12
M-9	1020	63.8	0.215	0.40	1.41	1605.15	0.01	0.433	1.18	0.01	0.242	1.10
M-4	1006	73.1	0.215	0.16	1.95	1863.27	0.01	0.433	1.28	0.01	0.242	1.16
M-5	1005	69.7	0.215	0.25	1.68	1883.46	0.01	0.433	1.23	0.01	0.242	1.13
M-6	963	74.4	0.215	0.14	2.08	3008.58	0.01	0.433	1.24	0.00	0.242	1.13
M-7	938	74.8	0.215	0.10	2.34	4037.70	0.01	0.433	1.23	0.00	0.242	1.13
I-3	1005	62.4	0.215	0.46	1.34	1883.46	0.01	0.433	1.15	0.01	0.242	1.08
I-5	964	76.2	0.215	0.12	2.19	2974.12	0.01	0.433	1.25	0.00	0.242	1.14
S-6	1023	65	0.215	0.33	1.51	1555.32	0.01	0.433	1.21	0.01	0.242	1.12
S-3	1005	63.6	0.215	0.37	1.46	1883.46	0.01	0.433	1.18	0.01	0.242	1.10
S-5	1006	66.8	0.215	0.30	1.56	1863.27	0.01	0.433	1.21	0.01	0.242	1.12

Note: *β*_Li_: Li diffusional fractionation factor is from Richter, *et al.*^41^. NBO/T_si-melt_: the ratio of NBO (non-bridging oxygen) to T (tetrahedron) of the Si-melt. K_Mg_: partition coefficient of Li between two immiscible silicates melt. K_Li_ = 1.0091(NBO/T_si-melt_)^−0.364^ is obtained by fitting experimental data of Veksler, *et al.*^54^. D_Li_/D_si_: ratio of diffusivity of Li over Si in basaltic melt is from Zhang, *et al.*^55^. α _(1/2+1/2)_: calculated α value with the bulk melt = C_∞ (1/2+1/2)_. *γ*1: calculated *γ* value using α _(1/2+1/2)_. δ^7^Li _(1/2+1/2)_: calculated δ^7^Li with the bulk melt = C_∞ (1/2+1/2)_, and initial δ^7^Li = 1 is assumed. α _(3/3+1/3)_: calculated α value with the bulk melt = C_∞ (2/3+1/3)_. *γ*2: calculated *γ* value using α _(3/3+1/3)_. δ^7^Li _(2/3+1/3)_: calculated δ^7^Li with the bulk melt = C_∞ (2/3+1/3)_, and initial δ^7^Li = 1 is assumed.
