# Supplementary material for: Non-traditional stable isotope behaviors in immiscible silica-melts in a mafic magma chamber
Source: Sci Rep. 2015 Dec 1;5:17561. doi: 10.1038/srep17561 (PMC4665163; doi:10.1038/srep17561)
Supplement: Supplementary Information [file srep17561-s1.pdf]

Non-traditional stable isotope behaviors in immiscible silica-melts in a mafic magma chamber

Dan Zhu [a*], Huiming Bao[a, b], and Yun Liu [a]

[a] *State Key Laboratory of Ore Deposit Geochemistry, Institute of Geochemistry, Chinese Academy of Sciences, Guiyang 550081, China*

[b] *Department of Geology and Geophysics, E235 Howe-Russell Complex, Louisiana State University, Baton Rouge, LA 70803, USA*

* Corresponding author: Dan Zhu (zhudan@vip.gyig.ac.cn)

Supplementary information

Table S1: compiled Fe isotope data for granitoids

**A-Type Granitoids**				
**Sample**	**SiO_2**	**$\delta^{57}Fe$**	**2SE**	**Reference**
**EV9101**	76.80	0.58	0.05	Sossi et al., 2012
**CB9092**	77.00	0.45	0.05	Sossi et al., 2012
**PO9207**	76.10	0.38	0.05	Sossi et al., 2012
**9108**	77.70	0.36	0.05	Sossi et al., 2012
**STM-1 (USGS syenite)**	59.60	0.20	0.05	Sossi et al., 2012
**NSL (peralk. Rhyolite)**	75.50	0.44	0.05	Sossi et al., 2012
**AC-E**	70.35	0.49	0.05	Sossi et al., 2012
**82-QC-32C Peralkaline granite**	76.00	0.40	0.05	Sossi et al., 2012
**Coso #4/7**	77.00	0.34	0.05	Sossi et al., 2012
**Coso #5/4**	76.90	0.46	0.05	Sossi et al., 2012
**Coso # 14/3**	76.60	0.43	0.05	Sossi et al., 2012
**Coso # 20/5**	76.40	0.40	0.05	Sossi et al., 2012
**Coso # 25/6**	76.70	0.31	0.05	Sossi et al., 2012
**H1970-8**	73.10	0.28	0.05	Sossi et al., 2012
**BP9220**	75.81	0.30	0.05	Sossi et al., 2012
**Granite, Watergums**	73.6	0.227	0.034	Telus et al., 2012
**Granite, Mumbella**	77	0.467	0.032	Telus et al., 2012
**L80-44**	77.62	0.43	0.05	Foden et al., 2015
**M3-86**	75.66	0.32	0.03	Foden et al., 2015
**M3-79**	74.99	0.32	0.04	Foden et al., 2015
**M3-53**	74.11	0.36	0.06	Foden et al., 2015
**6YC-143**	76.93	0.31	0.03	Foden et al., 2015
**6YC-137**	76.88	0.50	0.03	Foden et al., 2015
**I-463**	75.25	0.35	0.03	Foden et al., 2015
**L74-14G**	76.85	0.37	0.06	Foden et al., 2015
**KW08-15**	72.66	0.36	0.05	Foden et al., 2015
**KW08-1**	75.02	0.25	0.05	Foden et al., 2015
**MG3**	71.43	0.22	0.01	Foden et al., 2015
**JF07-117**	74.50	0.29	0.02	Foden et al., 2015
**JF07-116**	74.42	0.33	0.06	Foden et al., 2015
**HS018**	62.88	0.36	0.03	Foden et al., 2015
**HS020**	58.75	0.38	0.05	Foden et al., 2015
**194030**	68.51	0.44	0.01	Foden et al., 2015
**CB-91-4**	63.8	0.25	0.06	Zambardi et al., 2014
**CB-91-7**	55.9	0.12	0.05	Zambardi et al., 2014
**CB-91-13**	67.5	0.18	0.02	Zambardi et al., 2014

**CB-91-21**	75.3	0.54	0.06	Zambardi et al., 2014
**CB-91-32**	71.5	0.45	0.09	Zambardi et al., 2014
**CB-91-48**	61.8	0.1	0.04	Zambardi et al., 2014
**CB-91-DI5**	58.3	0.13	0.05	Zambardi et al., 2014
**CB-91-DI1R**	73.2	0.34	0.04	Zambardi et al., 2014
**BSB-01(BSB)**	76.2	0.62	0.06	Zambardi et al., 2014
**AC1-016**	75.05	0.48	0.06	Zambardi et al., 2014
**AC1-79**	69.93	0.3	0.06	Zambardi et al., 2014
**AC1-134**	62.68	0.15	0.06	Zambardi et al., 2014
**AC1-178**	67.12	−0.09	0.06	Zambardi et al., 2014
**I-Type Granitoids**				
**Sample**	**SiO_2**	**$\delta^{57}Fe$**	**2SE**	**Reference**
**PC9401**	64.90	0.17	0.05	Sossi et al., 2012
**Ga**	69.90	0.14	0.05	Sossi et al., 2012
**GSR-1**	72.80	0.24	0.05	Sossi et al., 2012
**GRA**	62.80	0.08	0.05	Sossi et al., 2012
**GS-N (CRPG granite)**	65.99	0.18	0.05	Sossi et al., 2012
**SEG 03 01 Rhyolite**	70.43	0.12	0.05	Sossi et al., 2012
**SEG 03 03 Dacitic lava flow**	67.88	0.06	0.05	Sossi et al., 2012
**SEG 03 43 Rhyodacite**	68.66	0.10	0.05	Sossi et al., 2012
**SEG 03 44 Dacitic ash flow tuff**	64.51	0.01	0.05	Sossi et al., 2012
**SB 87 56 Rhyolitic lava flow**	70.05	-0.06	0.05	Sossi et al., 2012
**SEG 03 31 Rhyolite**	71.39	0.10	0.05	Sossi et al., 2012
**PU 03 27**	66.47	0.07	0.05	Sossi et al., 2012
**PU 02 29**	69.53	0.12	0.05	Sossi et al., 2012
**K-22 Rhyolite lava, Novarupta dome**	76.60	0.15	0.05	Sossi et al., 2012
**K-45 Dacite pumice, layer C**	64.60	-0.03	0.05	Sossi et al., 2012
**Q82J-13 Granite**	71.90	0.15	0.05	Sossi et al., 2012
**82-QC-44 Granodiorite**	65.80	0.13	0.05	Sossi et al., 2012
**Q83J-99 Granite**	76.91	0.21	0.05	Sossi et al., 2012
**Q83J-101 Granodiorite**	68.40	0.00	0.05	Sossi et al., 2012
**Q82J-9 Porphyry (rhyolite) dike**	74.20	0.13	0.05	Sossi et al., 2012
**Bona1 -Gdrt**	66.30	0.16	0.05	Sossi et al., 2012
**Iorio1**	62.39	0.15	0.05	Sossi et al., 2012
**H5-A**	72.70	0.23	0.05	Sossi et al., 2012
**H4-5**	72.00	0.26	0.05	Sossi et al., 2012
**H4-7**	72.60	0.21	0.05	Sossi et al., 2012
**H4-3**	66.30	0.03	0.05	Sossi et al., 2012
**H2**	68.60	0.15	0.05	Sossi et al., 2012
**H3-Haf**	69.60	0.15	0.05	Sossi et al., 2012
**H1104-A**	68.40	0.16	0.05	Sossi et al., 2012

**HZ-A**	63.90	0.09	0.05	Sossi et al., 2012
**Hek-8**	64.70	0.08	0.05	Sossi et al., 2012
**G97-18**	69.79	0.19	0.05	Sossi et al., 2012
**G99-22**	66.92	0.02	0.05	Sossi et al., 2012
**RGM-1**	73.40	0.29	0.05	Sossi et al., 2012
**GSP-2**	66.60	0.23	0.05	Sossi et al., 2012
**GBW-7111**	59.68	0.16	0.05	Sossi et al., 2012
**Tonalite, Tuross Head**	60.13	0.184	0.026	Telus et al., 2012
**Granodiorite, Yurammie**	65.27	0.12	0.038	Telus et al., 2012
**Granodiorite, Glenbog**	67.39	0.167	0.028	Telus et al., 2012
**Adamellite, Wallagaraugh**	74.87	0.287	0.03	Telus et al., 2012
**Tonalite, Jindabyne**	62.29	0.129	0.026	Telus et al., 2012
**Qtz. Diorite, Clear Hills**	56.2	0.109	0.03	Telus et al., 2012
**Adamellite, Eugowra**	69.51	0.194	0.041	Telus et al., 2012
**Adamellite, Eugowra, Lysterfield**	63.73	0.193	0.025	Telus et al., 2012
**Dacite, Kadoona**	66.17	0.147	0.031	Telus et al., 2012
**L81-25**	69.39	-0.06	0.07	Foden et al., 2015
**BOF**	73.18	0.30	0.07	Foden et al., 2015
**I-1208**	70.58	0.15	0.14	Foden et al., 2015
**I-841**	73.29	0.24	0.01	Foden et al., 2015
**X-37**	75.46	0.26	0.05	Foden et al., 2015
**I-459**	73.85	0.28	0.04	Foden et al., 2015
**I-1001**	73.88	0.31	0.03	Foden et al., 2015
**A1109/5**	71.95	0.16	0.02	Foden et al., 2015
**A1109/14**	62.16	0.13	0.02	Foden et al., 2015
**SJF7**	68.90	0.17	0.02	Foden et al., 2015
**A1109 11**	73.66	0.24	0.06	Foden et al., 2015
**S-Type Granitoids**				
**Granodiorite, Cooma**	72	0.133	0.034	Telus et al., 2012
**Granodiorite, Cowra**	67.87	0.114	0.029	Telus et al., 2012
**Granodiorite, Jillamatong**	67.68	0.15	0.029	Telus et al., 2012
**Adamellite, Minnegans**	68.72	0.215	0.059	Telus et al., 2012
**Granodiorite, Dalgety**	68.21	0.182	0.026	Telus et al., 2012
**Adamellite, Numbla**	73.48	0.185	0.038	Telus et al., 2012
**Adamellite, Numbla, Koetong**	73.65	0.17	0.032	Telus et al., 2012
**Granite, Strathbogie**	73.65	0.219	0.025	Telus et al., 2012
**Dacite, Hawkins**	68.05	0.122	0.03	Telus et al., 2012
**L80-59**	76.00	0.39	0.03	Foden et al., 2015
**I-569**	76.71	0.40	0.04	Foden et al., 2015
**SS2000-11**	76.28	0.16	0.01	Foden et al., 2015
**90-PE1**	75.04	0.28	0.03	Foden et al., 2015

**91-CYH1**	72.09	0.37	0.04	Foden et al., 2015
**99220043**	70.28	0.25	0.05	Foden et al., 2015
**2001220071**	76.28	0.32	0.01	Foden et al., 2015
**99220045**	75.18	0.29	0.04	Foden et al., 2015
**2001220070**	76.12	0.34	0.07	Foden et al., 2015
**61692**	73.32	0.45	0.02	Foden et al., 2015
**137471**	70.90	0.29	0.06	Foden et al., 2015
**RB001**	70.51	0.43	0.01	Foden et al., 2015
**RB002**	74.49	0.27	0.09	Foden et al., 2015
**RB003**	71.23	0.29	0.09	Foden et al., 2015
**RB004**	70.10	0.38	0.07	Foden et al., 2015

Table S2: compiled Mg isotope data for granitoids

**A-Type Granitoids**				
**Sample**	**SiO_2**	**$\delta^{26}Mg$**	**SE**	**Reference**
**9715-1e**	74.1	-0.04	0.11	Li et al., 2010
**9717-1**	73	-0.1	0.08	Li et al., 2010
**9718-1**	72.8	-0.08	0.08	Li et al., 2010
**9767-1e**	77	-0.21	0.11	Li et al., 2010
**9801-2e**	75.7	0.12	0.09	Li et al., 2010
**9832-2**	76.3	-0.18	0.06	Li et al., 2010
**9849-1**	74.4	0.01	0.1	Li et al., 2010
**DW-2**	72.9	0.28	0.1	Li et al., 2010
**DW-3**	74.5	0.18	0.07	Li et al., 2010
**9757-3e**	75.9	-0.18	0.09	Li et al., 2010
**9757-4**	76.2	-0.12	0.08	Li et al., 2010
**9780-1e**	76.2	0.17	0.09	Li et al., 2010
**9781-1e**	76.3	0.27	0.09	Li et al., 2010
**9781-4e**	75.8	0.34	0.09	Li et al., 2010
**9781-5e**	76.8	0.18	0.09	Li et al., 2010
**9782-1e**	76.6	-0.01	0.09	Li et al., 2010
**Baishi-1**	76.1	-0.12	0.06	Li et al., 2010
**9843-1**	74.9	-0.1	0.07	Li et al., 2010
**9843-6e**	75.7	-0.28	0.09	Li et al., 2010
**A1**	73.6	-0.21	0.092	Telus et al., 2012
**A2**	77	0.026	0.092	Telus et al., 2012
**I-Type Granitoids**				
**MG14**	67.2	-0.15	0.09	Li et al., 2010

**MG20**	74.9	-0.24	0.09	Li et al., 2010
**AB40**	68.8	-0.18	0.09	Li et al., 2010
**AB105**	67.9	-0.17	0.09	Li et al., 2010
**AB128**	64.5	-0.24	0.06	Li et al., 2010
**AB249**	66.2	-0.16	0.09	Li et al., 2010
**AB289**	73.8	-0.21	0.08	Li et al., 2010
**AB293**	63.6	-0.2	0.07	Li et al., 2010
**I4**	74.87	-0.446	0.092	Telus et al., 2012
**I8**	63.73	-0.216	0.092	Telus et al., 2012
**07LD-1**	69.2	-0.26	0.04	Liu et al., 2010
**07LD-2**	68.5	-0.22	0.05	Liu et al., 2010
**07ZB-1**	68.6	-0.15	0.04	Liu et al., 2010
**07ZB-6**	65.6	-0.18	0.04	Liu et al., 2010
**07FJ-2**	70.3	-0.26	0.04	Liu et al., 2010
**07FJ-6**	68.1	-0.25	0.05	Liu et al., 2010
**07DT-3**	67.3	-0.2	0.03	Liu et al., 2010
**YFD-8**	68.7	-0.14	0.03	Liu et al., 2010
**07MC-1**	65.9	-0.24	0.03	Liu et al., 2010
**07MC-3**	65.4	-0.22	0.04	Liu et al., 2010
**07MC-6**	66.8	-0.24	0.04	Liu et al., 2010
**S-Type Granitoids**				
**VB30**	66.1	-0.23	0.07	Li et al., 2010
**VB98**	73.5	-0.14	0.1	Li et al., 2010
**S4**	68.72	-0.251	0.092	Telus et al., 2012
**S6**	73.48	-0.201	0.092	Telus et al., 2012
**S8**	73.65	-0.209	0.092	Telus et al., 2012

Table S3: compiled Si isotope data for granitoids

**A-Type Granitoids**				
**Sample**	**SiO_2**	**$\delta^{30}Si$**	**SE**	**Reference**
**2001**	73.9	-0.13	0.1	Savage et al., 2012
**PG-11**	75.1	-0.15	0.06	Savage et al., 2012
**2000**	74.6	-0.19	0.09	Savage et al., 2012
**CB-91-4**	63.8	-0.22	0.01	Zambardi et al., 2014
**CB-91-7**	55.9	-0.25	0.02	Zambardi et al., 2014
**CB-91-13**	67.5	-0.19	0.04	Zambardi et al., 2014
**CB-91-21**	75.3	-0.14	0.03	Zambardi et al., 2014
**CB-91-32**	71.5	-0.11	0.04	Zambardi et al., 2014

**CB-91-48**	61.8	-0.16	0.03	Zambardi et al., 2014
**CB-91-DI5**	58.3	-0.24	0.03	Zambardi et al., 2014
**CB-91-DI1R**	73.2	-0.12	0.03	Zambardi et al., 2014
**BSB-01(BSB)**	76.2	-0.1	0.03	Zambardi et al., 2014
**AC1-016**	75.05	-0.16	0.04	Zambardi et al., 2014
**AC1-79**	69.93	-0.19	0.03	Zambardi et al., 2014
**AC1-134**	62.68	-0.2	0.04	Zambardi et al., 2014
**AC1-178**	67.12	-0.31	0.04	Zambardi et al., 2014
**I-Type Granitoids**				
**AB006**	67.2	-0.18	0.06	Savage et al., 2012
**AB082**	66.9	-0.21	0.1	Savage et al., 2012
**AB102**	72.2	-0.18	0.11	Savage et al., 2012
**AB195**	65.3	-0.19	0.07	Savage et al., 2012
**AB234**	69.2	-0.24	0.1	Savage et al., 2012
**MG58**	72.5	-0.14	0.09	Savage et al., 2012
**BB10**	71.2	-0.18	0.04	Savage et al., 2012
**BB21**	76.6	-0.15	0.14	Savage et al., 2012
**BB87**	67.7	-0.25	0.14	Savage et al., 2012
**G38**	68	-0.14	0.08	Savage et al., 2012
**S-Type Granitoids**				
**BB02**	73.5	-0.23	0.14	Savage et al., 2012
**BB09**	68.2	-0.26	0.14	Savage et al., 2012
**BB12**	67.4	-0.25	0.04	Savage et al., 2012
**BB53**	67.8	-0.33	0.15	Savage et al., 2012
**BB83**	67.4	-0.22	0.13	Savage et al., 2012
**VB140**	72.5	-0.37	0.07	Savage et al., 2012
**NEB247**	72.7	-0.11	0.16	Savage et al., 2012

Table S4: compiled Li isotope data for granitoids

**A-Type Granitoids**				
**Sample**	**SiO_2**	**δ^7Li**	**SE**	**Reference**
9715-1	74.1	2.8	1	Li et al., 2010
9717-1	73	3.2	1	Li et al., 2010
9718-1	72.8	0.8	1	Li et al., 2010
9767-1	77	0.8	1	Li et al., 2010
9801-2	75.7	6.9	1	Li et al., 2010
9832-2	76.3	3.1	1	Li et al., 2010
9849-1	74.4	5.1	1	Li et al., 2010

DW-2	72.9	-1.8	1	Li et al., 2010
DW-3	74.5	2.9	1	Li et al., 2010
9757-3	75.9	0.7	1	Li et al., 2010
9757-4	76.2	1.2	1	Li et al., 2010
9780-1	76.2	4.6	1	Li et al., 2010
9781-1	76.3	0.7	1	Li et al., 2010
9781-4	75.8	0.5	1	Li et al., 2010
9781-5	76.8	1.5	1	Li et al., 2010
9782-1	76.6	1.7	1	Li et al., 2010
Baishi-1	76.1	2.4	1	Li et al., 2010
9843-1	74.9	1.1	1	Li et al., 2010
9843-6	75.7	3.2	1	Li et al., 2010
JH-10	57.5	3.1	1	Teng et al., 2009
JH-11	58.9	-1.2	1	Teng et al., 2009
JH-13	60.2	0.4	1	Teng et al., 2009
JH-14	56	0.3	1	Teng et al., 2009
JH-15-2	62.6	-1	1	Teng et al., 2009
JH-17-2	61.4	1.8	1	Teng et al., 2009
JH-21	67	2.8	1	Teng et al., 2009
JH-22	59.9	1.7	1	Teng et al., 2009
JH-25	62.9	-3.2	1	Teng et al., 2009
JH-30	60	-2.2	1	Teng et al., 2009
JH-09	70.84	2.1	1	Teng et al., 2009
JH-12	71.33	2.1	1	Teng et al., 2009
JH-15-1	71.16	0.9	1	Teng et al., 2009
JH-17-1	70.49	0.2	1	Teng et al., 2009
JH-26	70.28	1.7	1	Teng et al., 2009
JH-27	69.67	1.8	1	Teng et al., 2009
JH-28	68.9	0.9	1	Teng et al., 2009
JH-29	69.1	2.9	1	Teng et al., 2009
JH-31	66.75	3	1	Teng et al., 2009
**I-Type Granitoids**				
MG14	67.2	0.3	1.00	Li et al., 2010
MG20	74.9	0.8	1.00	Li et al., 2010
AB40	68.8	-2.5	1.00	Li et al., 2010
AB105	67.9	-2.1	1.00	Li et al., 2010
AB128	64.5	0.1	1.00	Li et al., 2010
AB249	66.2	2.7	1.00	Li et al., 2010
AB289	73.8	1.8	1.00	Li et al., 2010
AB293	63.6	-0.1	1.00	Li et al., 2010
JG-7	70.74	4.7	0.60	2007Marks et al. CG

JG-6	71.517	3.9	0.60	2007Marks et al. CG
JG-5	70.678	4.2	0.60	2007Marks et al. CG
NEB215	61.59	3.6	1.20	2004Bryant et al. TRSE
NEB212	64.63	2	0.20	2004Bryant et al. TRSE
NEB197	68.01	2.5	1.30	2004Bryant et al. TRSE
NEB232	68.74	4	0.30	2004Bryant et al. TRSE
NEB 414	68.5	0.2		2004Bryant et al. TRSE
NEB54	70.64	1.8	0.80	2004Bryant et al. TRSE
NEB200	67.07	0.5		2004Bryant et al. TRSE
MNG432	57.52	2.9	1.20	2004Bryant et al. TRSE
MNG455	66.64	4.3	1.30	2004Bryant et al. TRSE
MNG223	75.3	2.8	1.40	2004Bryant et al. TRSE
NEB482	64.33	2.3	1.10	2004Bryant et al. TRSE
NEB208	70.3	3.1	0.40	2004Bryant et al. TRSE
72056	65.52	2.1	0.40	2004Bryant et al. TRSE
72332	60.48	8.1	0.50	2004Bryant et al. TRSE
72341	64.36	3.3		2004Bryant et al. TRSE
72016	67.85	6.2		2004Bryant et al. TRSE
72009	67.63	4.5	1.80	2004Bryant et al. TRSE
72098	61.74	2.7	0.50	2004Bryant et al. TRSE
72101	55.71	4.8	0.70	2004Bryant et al. TRSE
72151	65.84	3.1	0.20	2004Bryant et al. TRSE
MG14	67.18	0.3	1.00	2004Teng et al., GCA
MG20	74.91	0.8	1.00	2004Teng et al., GCA
AB40	68.77	-2.5	1.00	2004Teng et al., GCA
AB105	67.86	-2.1	1.00	2004Teng et al., GCA
AB128	64.47	0.1	1.00	2004Teng et al., GCA
AB249	66.18	2.7	1.00	2004Teng et al., GCA
AB289	73.79	1.8	1.00	2004Teng et al., GCA
AB293	63.6	-0.1	1.00	2004Teng et al., GCA
**S-Type Granitoids**				
VB30	66.1	-1.4	1	Li et al., 2010
VB98	73.5	-1.1	1	Li et al., 2010
3-1B	75.06	4	1	2006Teng et al. AM
4-1	71.98	6.3	1	2006Teng et al. AM
1-1	74.72	2.1	1	2006Teng et al. AM
2-1	74.2	0.2	1	2006Teng et al. AM
HP-3B	73.9	1.6	1	2006Teng et al. AM
HP-8 4L		1.1	1	2006Teng et al. AM
HP-8 8L	68.75	0	1	2006Teng et al. AM
HP-20	74.96	2.9	1	2006Teng et al. AM

HP-1	73.78	-3.1	1	2006Teng et al. AM
HP-2	71.7	-2.1	1	2006Teng et al. AM
HP-6	72.89	-1.47	1	2006Teng et al. AM
HP-14	73.2	-1.4	1	2006Teng et al. AM
HP2A	73.8	2.2	1	2006Teng et al. AM
HP10B	74.6	0.3	1	2006Teng et al. AM
HP13A	74.4	2.17	1	2006Teng et al. AM
HP13C	74.4	2.3	1	2006Teng et al. AM
HP14A	72.9	2	1	2006Teng et al. AM
HP17	75.4	-1.08	1	2006Teng et al. AM
HP22	75.2	-0.1	1	2006Teng et al. AM
HP24B	73.1	3.9	1	2006Teng et al. AM
HP30A	69.8	5.49	1	2006Teng et al. AM
HP39A	72.2	6.55	1	2006Teng et al. AM
HP43A	72.6	0.02	1	2006Teng et al. AM
HP44A	72.7	1.05	1	2006Teng et al. AM
HP45B	74.2	0.97	1	2006Teng et al. AM
MNG459	72.46	2.4	1·0	2004Bryant et al. TRSE
MNG419	69.52	2.4	1·0	2004Bryant et al. TRSE
NEB247	72.66	1.5	0·2	2004Bryant et al. TRSE
NEB251	73.58	-0.1	0.3	2004Bryant et al. TRSE
NEB246	70.42	2.8	0·6	2004Bryant et al. TRSE
NEB219	73.35	2.7	1·1	2004Bryant et al. TRSE
NEB290	68.71	1.2	0·6	2004Bryant et al. TRSE
NEB294	69.51	1.9	2·6	2004Bryant et al. TRSE
NEB484	66.87	0.9	0·8	2004Bryant et al. TRSE
NEB475	67.95	0.5	0·6	2004Bryant et al. TRSE
NEB 415	66.53	0.9	1·3	2004Bryant et al. TRSE
VB30	66.06	-1.4	1	2004Teng et al., GCA
VB98	73.49	-1.1	1	2004Teng et al., GCA

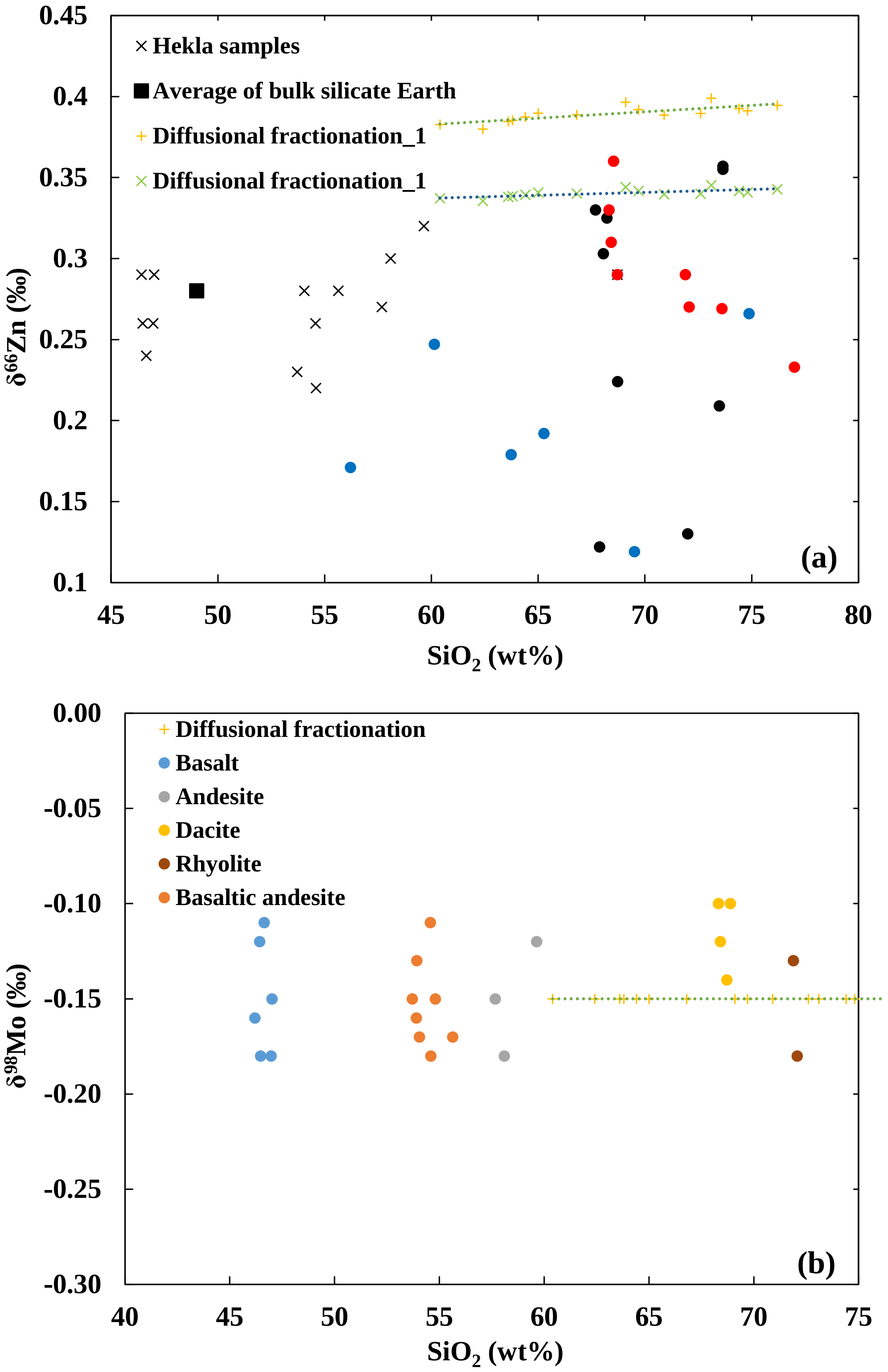

Fig. S1 : Observed and predicted stable isotope compositions of granitoids. (a) Zn isotopes from Chen et al., 2013 and references. The diffusional fractionation trend is calculated using Eq. (9). (b) Mo isotopes from Yang et al., 2015. The diffusional fractionation trend is calculated using Eq. (9).
